# Cortical Thickness and Behavior Abnormalities in Children Born Preterm

**DOI:** 10.1371/journal.pone.0042148

**Published:** 2012-07-30

**Authors:** Leire Zubiaurre-Elorza, Sara Soria-Pastor, Carme Junque, Roser Sala-Llonch, Dolors Segarra, Nuria Bargallo, Alfons Macaya

**Affiliations:** 1 Department of Psychiatry and Clinical Psychobiology, Faculty of Medicine, University of Barcelona, Barcelona, Spain; 2 Institut d’Investigacions Biomèdiques August Pi i Sunyer (IDIBAPS), Barcelona, Spain; 3 Clinical Institute of Neurosciences, Hospital Clínic Barcelona, Barcelona, Spain; 4 Neuroradiology Section, Radiology Department, Centre de Diagnòstic per la Imatge (CDIC), Hospital Clínic, Barcelona, Spain; 5 Grup de Recerca en Neurologia Pediàtrica, Institut de Recerca Vall d’Hebron, Autonomous University of Barcelona, Barcelona, Spain; Centre Hospitalier Universitaire Vaudois Lausanne - CHUV, UNIL, Switzerland

## Abstract

**Aim:**

To identify long-term effects of preterm birth and of periventricular leukomalacia (PVL) on cortical thickness (CTh). To study the relationship between CTh and cognitive-behavioral abnormalities.

**Methods:**

We performed brain magnetic resonance imaging on 22 preterm children with PVL, 14 preterm children with no evidence of PVL and 22 full-term peers. T1-weighted images were analyzed with FreeSurfer software. All participants underwent cognitive and behavioral assessments by means of the Wechsler Intelligence Scales for Children-Fourth Edition (WISC-IV) and the Child Behavior Checklist (CBCL).

**Results:**

We did not find global CTh differences between the groups. However, a thinner cortex was found in left postcentral, supramarginal, and caudal middle rostral gyri in preterm children with no evidence of PVL than in the full-term controls, while PVL preterm children showed thicker cortex in right pericalcarine and left rostral middle frontal areas than in preterm children with no evidence of PVL. In the PVL group, internalizing and externalizing scores correlated mainly with CTh in frontal areas. Attentional scores were found to be higher in PVL and correlated with CTh increments in right frontal areas.

**Interpretation:**

The preterm group with no evidence of PVL, when compared with full-term children, showed evidence of a different pattern of regional thinning in the cortical gray matter. In turn, PVL preterm children exhibited atypical increases in CTh that may underlie their prevalent behavioral problems.

## Introduction

Neonates born preterm have a high risk of developing long-term psychiatric and behavioral disorders [1]–[3]. The degree to which prematurity itself or associated cerebral lesions, i.e. the encephalopathy of prematurity, may account for this elevated risk, is currently unknown. Specifically, preterm birth has been related to attention/hyperactivity symptoms [4]–[6], a high prevalence of autism [7], and higher scores in other behavioral disorders [8]–[10].

Normal development in childhood is accompanied by an increase in cortical thickness (CTh) in almost all brain areas, whereas there is a progressive cortical thinning during adolescence [11]. Studies in neurodevelopmental disorders have reported both thicker and thinner cortical regions. For instance, cortical thickening has been described in autism [12], [13], whereas cortical thinning has been found in attention deficit hyperactivity disorder [14].

Several studies have reported global and regional differences in brain tissue volumes in preterm children [15], [16] and PVL preterm cohorts [17]–[19]. However, there have been only two studies in adolescent subjects where the effect of preterm birth on CTh is investigated. Martinussen et al [20] found different areas of CTh abnormalities (both increments and decrements) at a mean age of 15 years in very low birth-weight and small for gestational age preterm subsamples. Moreover, the study of Nagy et al [21] found similar results in preterm adolescents after controlling for age at evaluation. Since CTh is subject to dynamic changes during normal development, it is conceivable that delayed or altered development in premature children with PVL might result in a distinct pattern of CTh. Conversely, previous studies in preterm samples have not characterized the effects of PVL on CTh.

The aim of the current study was to investigate the effect of prematurity and PVL on CTh in childhood and the putative impact of the latter on cognition and behavior. Our hypothesis is that preterm birth and brain injury such as PVL could have an impact on CTh and that these cortical abnormalities may be related to cognitive-behavioral outcomes at school age.

## Methods

### Participant Characteristics

**Table 1:** Characteristics of the samples: neonatal and demographic data.

	PVL-Preterm n = 22	Preterm n = 14	Term n = 22	*Statistics* (*P*)
***Neonatal data***				
GA, mean ± SD, wk	30.2±2.9	31.9±1.1	39.5±1.0	132.12 (<.001)* ^,^ †
Birth weight, mean ± SD, g	1546±539	1701±474	3392±357	102.88 (<.001)* ^,^ †
Length at birth, mean ± SD, cm‡	39.5±3.9	42.4±4.5	50.7±2.1	49.35 (<.001)* ^,^ †
Head circumference, mean ± SD, cm↑	27.4±2.7	29.8±2.6	35.2±1.1	53.24 (<.001)* ^,^ †
Gender, male/female	13/9	10/4	14/8	0.56 (.754)▪
***Demographic data***				
Age at scan, mean ± SD, y	8.7±1.8	9.2±0.7	9.3±0.6	1.53 (225)*
Right-handed, n (%)	17 (77)	12 (86)	22 (100)	5.44 (.066)▪
Parental education, n				*8.85* (.065)▪
Low	9	2	3	
Intermediate	7	4	4	
High	6	8	15	

* *F* statistic.

† Significant differences found in term children compared with PVL-preterm and preterm children.

‡ Available data for length variable: 12 PVL-preterm, 14 preterm and 22 term children.

↑ Available data for head circumference variable: 12 PVL-preterm, 14 preterm and 18 term children.

▪ *X* ^2^ statistic.

The PVL group was recruited between June 2008 and May 2009 at the Hospital Universitari Vall d’Hebron (Barcelona, Spain) and consisted of 75 children (6–12 years of age) born preterm and with a history of PVL. Selection criteria included: gestational age (GA) <37 weeks, full intelligence quotient (FIQ) >70, neonatal diagnosis of PVL, and signs of PVL in the current MRI according to the criteria proposed by Flodmark et al [22]. The patients with PVL were recruited after being diagnosed with PVL in the neonatal period by means of ultrasound scans. This was defined as the presence of periventricular cysts of differing size and extension (PVL grades II–IV) or periventricular echodensities persisting over 14 days during the neonatal period. Neonatal peri/intraventricular hemorrhage grades III/IV or any other perinatal brain injury were exclusion criteria. From the 75 subjects, 21 were not available at the time of the study, 18 declined to participate, 11 were delivered at term, one did not show signs of PVL in the current MRI and two subjects had a FIQ <70. After applying these exclusion criteria, the sample comprised 22 PVL preterm participants. The clinical and magnetic resonance imaging (MRI) findings for this sample have previously been published [19].

Fourteen non-PVL preterm children, at low risk for neurodevelopmental deficits, were selected from a cohort followed at the Hospital Clinic (Barcelona, Spain). To be considered a low-risk preterm the following criteria were applied: 1) GA = 30–34 weeks, 2) birth weight below 2500 g, 3) Apgar score at fifth minute >7, 4) absence of major neonatal morbidity, including severe respiratory distress syndrome, mechanical ventilation, necrotizing enterocolitis, neonatal sepsis or bronchopulmonary dysplasia, and 5) absence of cerebral pathology on neonatal cranial ultrasound [16]. For the current study, the subcohort of low-risk preterms (non-PVL preterm children) was selected on the basis of their neonatal characteristics (GA and birth weight), which were group-mean matched with the PVL preterm sample. In addition, 22 infants delivered at term at the same center were enrolled as a full-term control group who were mainly friends and classmates of the preterm children. Exclusion criteria for all the groups included an FIQ equal to or less than 70, any history of brain injury and contraindications for an MRI scan. Table 1 summarizes the demographic and clinical characteristics of the sample. The highest education level of the parents was recorded as low, intermediate or high as in a previous preterm study conducted in adulthood [23]. The Ethics Committee of the University of Barcelona approved this study, and written informed parental consent was obtained for each infant.

### Neurodevelopmental Outcome Data

All participants were cognitively assessed with the Wechsler Intelligence Scales for Children - Fourth Edition (WISC-IV) [24]. Behavioral data were collected by means of the Child Behavior Checklist (CBCL) [25]. The checklist is a parent-report measure of children’s behavior. It is divided into the following subscales: Aggressive Behavior, Anxious/Depressed, Attention Problems, Delinquent Rule-Breaking Behavior, Social Problems, Somatic Complaints, Thought Problems, and Withdrawn. Some of these subscales group into two higher order factors – internalizing (withdrawn, somatic complaints and anxious/depressed scales) and externalizing (delinquent and aggressive behavior scales) – whose sum provides the total score.

### MRI Acquisition and Analysis

The three-dimensional MRI data sets were acquired at the *Centre de Diagnòstic per la Imatge* (Hospital Clinic, Barcelona, Spain). High-resolution T1-weighted images were acquired for all subjects on a 3-Tesla TIM TRIO scanner (Siemens, Erlangen, Germany). The following parameters were used: a MPRAGE sequence in sagittal orientation (TR/TE = 2300/2.98 ms; TI = 900 ms; 256×256 matrix, flip angle 9°, 1 mm^3^ isotropic voxel).

An automated cortical reconstruction of the T1-weighted images was performed by using FreeSurfer (version 4.3.1) software (http://surfer.nmr.mgh.harvard.edu). This method was used to create a three-dimensional cortical surface model of CTh using intensity and continuity information previously described in detail [26]. Processing of T1 high-resolution images included several procedures: removal of non-brain tissue [27], automated Talairach transformation, intensity normalization [28], tessellation of the gray matter/white matter boundary, automated topology correction, [29], [30] and surface deformation to detect gray matter/white matter and gray matter/cerebrospinal fluid boundaries [26]. Moreover, the cerebral cortex was divided into different regions according to gyral and sulcal structure information [31]. The resulting representation of cortical thickness was calculated as the distance between tissue boundaries (gray matter/white matter and gray matter/cerebrospinal fluid) [26].

### Statistical Analysis

The data were tested for normality and homogeneity. To do this, a multivariate analysis of variance was carried out to test the differences in the quantitative variables (demographic, neuropsychological, and behavioral variables) among the three groups. The chi-square test of independence was performed to analyze differences in proportions when appropriate. All statistical analyses were performed using SPSS v. 18.0 (SPSS Inc., Chicago, IL).

CTh maps were analyzed at the vertex-wise level separately for each hemisphere, using a general linear model approach as implemented by the FreeSurfer QDEC application. Before group analysis comparisons, individual CTh maps were registered to the standard template and smoothed with a Gaussian kernel of 15 mm FWHM. We performed t-test comparisons to evaluate CTh differences due to (1) prematurity itself (non-PVL preterm children versus full-term group), (2) PVL *per se* (PVL preterm group versus non-PVL sample), and (3) PVL plus prematurity (PVL preterm group versus full-term children). Moreover, we performed a correlation between the CTh and CBCL main indexes, the attention subscale, and FIQ. We adjusted CTh for age at scan in order to remove any variance in CTh associated with the effects of normal development and thereby avoid false positives and increase statistical power. Maps were corrected for multiple comparisons (family-wise error; FWE) using the Monte Carlo Null-Z simulation with 10,000 permutations. Global mean thickness and regional thickness values comprised in the clusters that were statistically significant in the CTh maps were obtained and tested in SPSS with age at evaluation as a covariate.

## Results

### Neonatal, Demographic, Cognitive, and Behavioral Data

Neonatal data are detailed in Table 1. The three groups were comparable for the purpose of independently evaluating the effects of PVL or preterm birth.

Although general intelligence mean scores were within the normal range for PVL preterm (92.32±14.2), normal preterm (106.8±13.8), and term groups (121.91±15.4), there were differences in FIQ among the three samples (F = 21.01; P<.001). Behavioral scores (Table 2) showed differences in PVL preterm children compared to full-term controls only in the attentional scale.

**Table 2:** CBCL mean scores: Parent reports of behavior.

	PVL-Preterm, Mean ± SD	Preterm, Mean ± SD	Term^a^, Mean ± SD	*F-Snedecor Statistic* (*P*)
CBCL Checklist				
Withdrawn	2.23±2.0	2.43±1.7	2.00±1.6	0.253 (.778)
Somatic complains	1.77±2.1	1.71±2.4	1.10±2.0	0.626 (.539)
Anxious/depressed	5.18±4.8	4.86±4.0	4.67±2.9	0.092 (.912)
Social problems	3.68±2.8	1.86±1.8	2.62±2.5	2.470 (.094)
Thought problems	1.36±1.6	0.50±0.8	0.67±1.0	2.695 (.077)
Attention problems	7.68±5.0	5.36±3.2	4.24±3.6	3.877 (.027)^b^
Delinquent behaviour	1.32±1.4	1.71±1.2	1.19±1.2	0.728 (.488)
Aggressive behaviour	8.82±5.5	8.36±5.7	8.14±4.9	0.090 (.914)
Total problems	32.05±20.0	27.21±16.1	24.62±14.6	1.023 (.366)
Internalizing problems	9.09±7.1	9.29±7.1	7.76±4.7	0.331 (.720)
Externalizing problems	10.14±6.6	10.07±6.5	9.33±5.6	0.105 (.901)

a Available data for 21 participants of the term group.

b Significant differences found in term children compared with PVL-preterm.

### Cortical Thickness Differences

Analysis of global CTh revealed no statistical differences between groups (left hemisphere: F = 0.71, P = .50; PVL preterm children: 2.91±0.15 mm; non-PVL preterm children: 2.87±0.08 mm; term children: 2.91±0.07 mm; and right hemisphere: F = 0.75, P = .50; PVL preterms: 2.86±0.15 mm; non-PVL preterms: 2.84±0.80 mm; term children: 2.88±0.07 mm).

### Prematurity and PVL Effects on Regional Cortical Thickness

**Figure 1:**
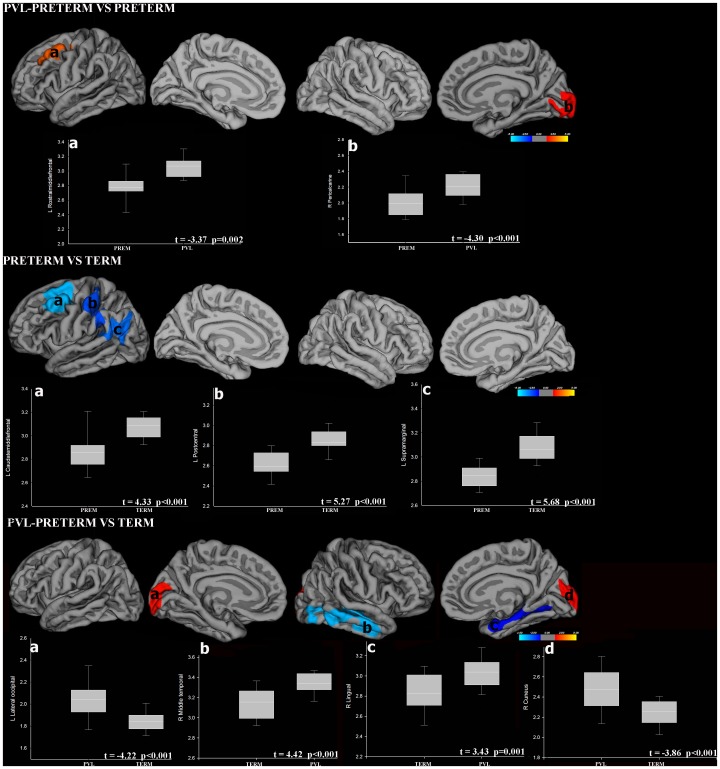
Maps of regional CTh comparisons between groups. (A) PVL preterm versus non-PVL preterm (B) Non-PVL preterm and (C) PVL preterm versus full-term children. Colour bars represent statistically significant (P<.05) thinning (blue) or thickening (yellow). Scatter plots show mean CTh values for each group and for illustration purposes have not been corrected for covariate effects.

Results from the t-test comparisons between groups are detailed in Table 3 and Figure 1. When comparing PVL preterm children to non-PVL preterm children a thicker cortex was observed, whereas when the same group was compared to their term peers, both regional thicker and thinner cortices were found bilaterally. When comparing non-PVL preterm sample to full-term children, regional decrements in CTh were found in specific areas of the left hemisphere.

**Table 3:** Regional differences in cortical thickness between groups.

Cluster size	Cluster Maxima Talairach Coordinates				P value	Side	Anatomical brain regions included in a cluster
	x		y	z			
**PVL-preterm > Non-PVL children**							
2409.61	−33.3	30.5		31.0	0.0023	Left	**Rostral middlefrontal**, caudal middle frontal, superior frontal
1549.51	10.7	−90.4		12.9	0.0410	Right	**Pericalcarine**, precuneus, cuneus, lingual
**Non-PVL preterm < Term children**							
2722.10	−41.9	16.6		41.6	0.0001	Left	**Caudal middle frontal**, superior frontal, rostral middle frontal, precentral
2260.64	−53.9	−48.9		24.6	0.0012	Left	**Supramarginal**, inferior parietal, lateral occipital, superior temporal, bankssts
1991.62	−45.0	−27.2		52.8	0.0037	Left	**Postcentral**, supramarginal, inferior parietal
**PVL-preterm vs Term children**							
1515.50	−10.4	−93.0		−4.6	0.0304	Left	**> Lateral occipital**, pericalcarine, cuneus, lingual
4005.74	55.4	−13.3		−16.5	0.0001	Right	**< Middle temporal,** inferior temporal, superior temporal, lateral occipital, bankssts,
1567.12	28.5	−47.8		−2.2	0.0390	Right	**< Lingual,** entorhinal, parahipocampal, fusiform
1549.15	7.5	−91.0		14.9	0.0411	Right	**> Cuneus,** pericalcarine, lateral occipital

Regions in bold represent the maximum coordinate encompassed in a given cluster.

Abbreviations: bankssts: banks of the superior temporal sulcus; > means thicker cortex and < thinner cortex.

Talaraich coordinates indicate: x increases from left (−) to right (+); y increases from posterior (−) to anterior (+); and z increases from inferior (−) to superior (+).

### Correlations between CTh, FIQ, and CBCL Scores

Regarding the general intelligence scores, a statistically significant negative correlation between FIQ and CTh was found only in the full-term group. Specifically, the maximum coordinates of the statistically significant clusters were located in the left superior frontal (cluster size  = 1372 mm^2^, cluster-level p value  = 0.0228, Talairach coordinates; x = −18.8, y = 59.4, z = 6.2) and right postcentral regions (cluster size  = 1458 mm^2^, cluster-level p value  = 0.020, Talairach coordinates; x = 52.9, y = −12.6, z = 44.2). However, the correlation did not achieve statistical significance either in the non-PVL preterm group or in the PVL preterm group.

**Figure 2:**
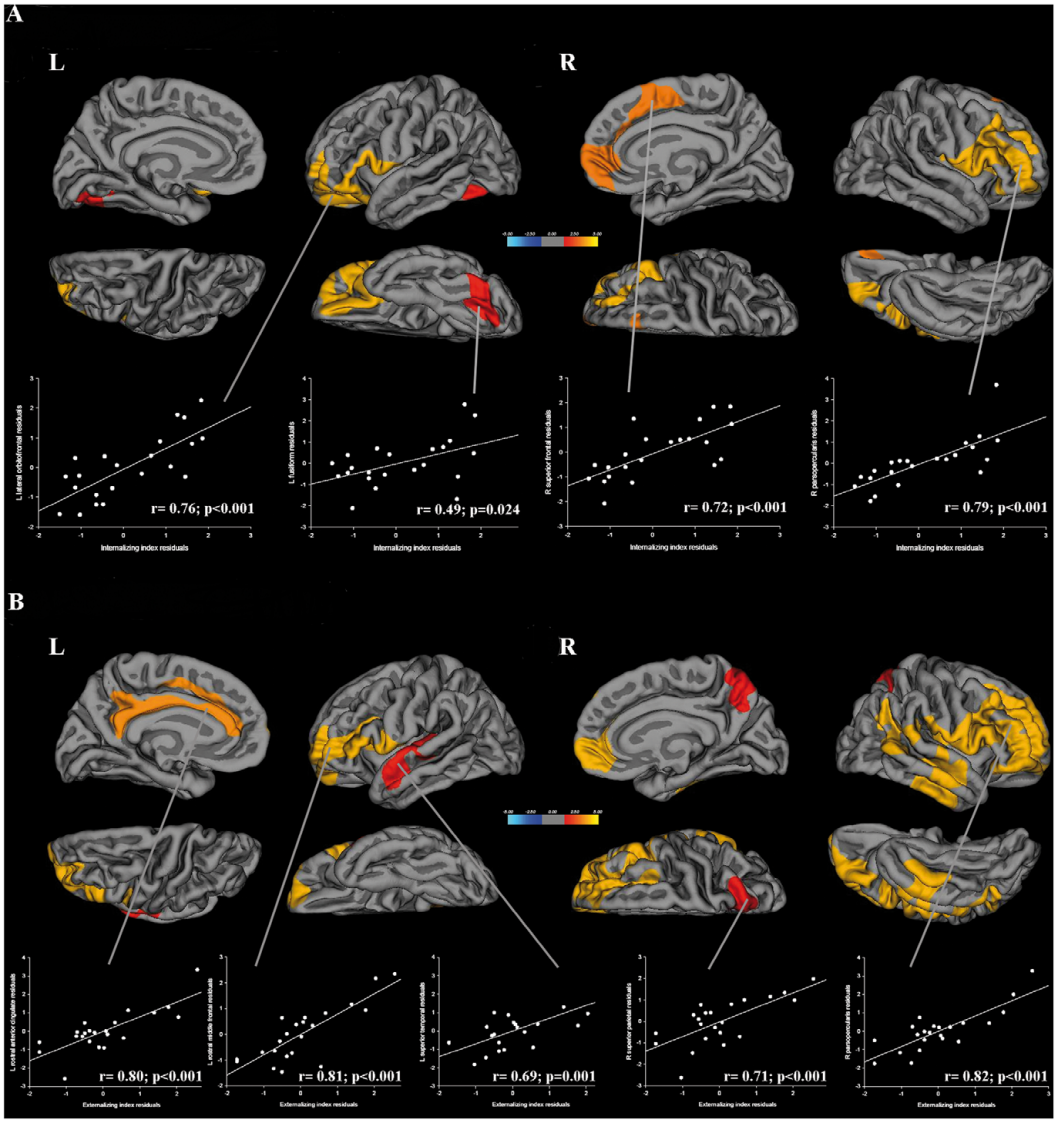
Correlation analysis between regional CTh and (A) internalizing or (B) externalizing indexes in the PVL-preterm group. The colour bar represents statistically significant (P<.05) thinning (blue) or thickening (yellow). Partial correlation statistics and scatter plots of mean CT in each area are displayed corrected for covariate effects.

**Figure 3:**
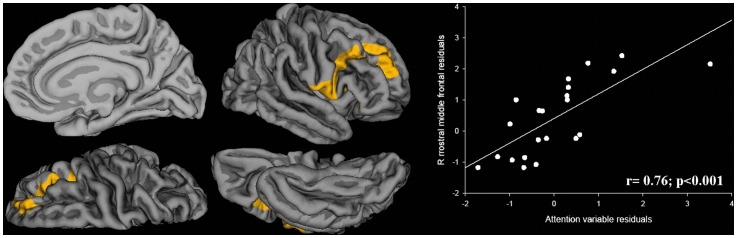
Map of correlation between attention subscale and CTh in PVL children. The colour bar represents statistically significant (P<.05) thinning (blue) or thickening (yellow). Partial correlation statistics and scatter plots of mean CT in each area are displayed corrected for covariate effects.

For the behavior measures, we found statistically significant positive correlations only within the PVL preterm group. The specific regions were associated with the three main indexes of CBCL – the internalizing, externalizing, and total scores are shown in Table 4. Figure 2 displays internalizing and externalizing scores and their respective correlations with regional CTh. No correlations were found between CTh and CBCL main index scores for the full-term and non-PVL preterm children. In PVL preterm children, attentional scores correlated positively with CTh in right frontal areas (Table 5 and Fig. 3).

**Table 4:** PVL sample; positive correlations between CTh and behavioural scores.

CBCL score	Side	ClusterSize(mm^2^)	Cluster-level*(P corrected)*	Local maxima Talairach coordinates			Anatomical brain regions included in a cluster
				x	y	z	
Internalizing score	L	4405.24	0.0001	−20.8	14.0	−17.5	**Lateral orbitofrontal**, medial orbitofrontal, rostral middle frontal, pars oorbitalis, pars triangularis, pars opercularis, precentral, postcentral
	L	1661.59	0.0240	−41.1	−69.0	−9.9	**Fusiform**, lingual, lateral occipital
	R	7065.47	0.0001	51.1	14.4	6.9	**Pars opercularis**, lateral orbitofrontal, rostral middle frontal, superior frontal, caudal middle frontal, insula, pars orbitalis, pars triangularis, precentral, postcentral
	R	2596.24	0.0007	8.8	18.0	43.1	**Superior frontal**, frontal pole, lateral orbitofrontal, medial orbitofrontal, rostral anterior cingulate, caudal anterior cingulate
Externalizing score	L	5207.94	0.0001	−32.8	51.0	−3.4	**Rostral middle frontal**, lateral orbitofrontal, caudal middle frontal, superior frontal, frontal pole, insula, pars opercularis, pars triangularis, precentral, postcentral
	L	3052.40	0.0004	−5.3	36.9	6.0	**Rostral anterior cingulate**, caudal anterior cingulate, posterior cingulate, isthmus cingulated, corpus callosum, precuneus, superior frontal
	L	1694.33	0.0211	−48.6	−5.8	−19.2	**Superior temporal**, transverse temporal, middle temporal
	R	18312.33	0.0001	37.1	12.0	20.2	**Pars opercularis**, lateral orbitofrontal, rostral middle frontal, superior frontal, caudal middle frontal, frontal pole, medial orbitofrontal, rostral anterior cingulate, inferior parietal, superior temporal, middle temporal, inferior temporal, bankssts, supramarginal, insula, pars orbitalis, pars triangularis, precentral, postcentral
	R	1566.03	0.0282	16.3	−53.5	60.3	**Superior parietal**, precuneus
Total score	L	3384.60	0.0001	−52.2	−25.8	18.2	**Supramarginal**, rostral middle frontal, insula, pars opercularis, pars triangularis, precentral, postcentral
	L	3105.87	0.0003	−5.2	37.0	6.6	**Rostral anterior cingulate**, caudal anterior cingulate, posterior cingulate, isthmus cingulated, corpus callosum, precuneus, superior frontal
	L	2136.23	0.0056	−33.3	51.0	−2.9	**Rostral middle frontal**, lateral orbitofrontal, superior frontal, frontal pole
	L	1526.02	0.0385	−40.7	−47.4	−10.5	Fusiform, inferior temporal, lateral occipital, lingual
	R	11735.78	0.0001	40.8	24.3	30.3	**Rostral middle frontal**, rostral anterior cingulate, caudal anterior cingulate, superior frontal, medial orbitofrontal, frontal pole, caudal middle frontal, supramarginal, insula, pars orbitalis, pars triangularis, parsopercularis, precentral, postcentral
	R	4857.96	0.0001	59.3	−11.7	−15.8	**Middle temporal**, inferior parietal, supramarginal, bankssts, superior temporal, inferior temporal
	R	1498.93	0.0481	7.7	−55.5	46.6	**Precuneus**, superior parietal
	R	1498.93	0.0481	7.7	−55.5	46.6	**Precuneus**, superior parietal

Regions in bold represent the maximum coordinate encompassed in a given cluster.

Abbreviations: bankssts: banks of the superior temporal sulcus, CBCL: Child Behavioural Checklist, L: left hemisphere, R: right hemisphere.

Talaraich coordinates indicate: x increases from left (−) to right (+); y increases from posterior (−) to anterior (+); and z increases from inferior (−) to superior (+).

**Table 5:** Statistically significant correlations between CTh and attention problems variable in PVL-preterm and Preterm groups.

CBCL index	Side		Cluster size (mm^2^)	Cluster-level *(P corrected)*		Local maxima Talairach coordinates			Anatomical brain regions included in a cluster
						x	y	z	
***PVL-preterm group (positive correlation)***									
Attention problems		R	3831.21		0.0001	40.5	25.1	30.5	**Rostral middle frontal**, caudal middle frontal, superior frontal, precentral, postcentral, insula, pars opercularis, pars triangularis, pars orbitalis

Regions in bold represent the maximum coordinate encompassed in a given cluster.

Abbreviations: CBCL: Child Behavioural Checklist, R: right hemisphere.

## Discussion

In this MRI study we documented that school-aged children with a history of preterm birth, with or without PVL, show abnormal patterns of CTh when compared with their control peers. Even though we did not find differences between groups in global CTh, our findings provide evidence that prematurity *per se* is associated with regional cortical thinning, whereas PVL resulted in specific areas of CTh thickening. Attentional scores were statistically higher in PVL children and correlated positively with right frontal thickness. Correlations between CTh and behavioral indexes were only observed in PVL preterm children. Finally, only the full-term group showed a significant negative correlation between FIQ and CTh.

The fact that regionally thicker and thinner cortical regions were found, is in agreement with previous studies in adolescents that used comparisons with typically developing brains [20], [21]. Martinussen et al. [20] studied a group of adolescents with a history of very low birth-weight and identified thinner parieto-temporo-occipital areas, as well as thicker fronto-occipital areas. In our study, we also found thinner cortex in the non-PVL preterm group involving extensive frontal and parietal regions and small areas in the temporal and occipital cortex. The cortical thinning is more restricted in our sample, probably because we used the Monte Carlo multiple comparisons correction. A similar preterm sample was analyzed by Nagy et al. [21], who observed thinner cortices in the parietal and temporal areas but thicker cortices in the inferior temporal gyrus and left ventrolateral prefrontal regions. We speculate that these decrements in CTh found in non-PVL preterm children might translate into a delayed maturation of some cortical areas, since these regions are supposed to reach their maximal thickness in childhood.^11^ The current preterm sample with no evidence of PVL is a subgroup of a sample used in a previous gray matter volumetric study [16]. There is a correspondence between current CTh findings and previous voxel-based morphometry results, in the sense that parietal and temporal areas showed both thinning and smaller volumes. However, there are other areas that showed volumetric reductions and did not show reductions in CTh. These discrepancies are probably due to the different neuroimaging approaches used in the two studies (voxel-based approach vs. vertex-based approach) and the fact that cerebral volume is a composite brain measure of both CTh and surface area, which may show reductions in preterm children.

No previous study has investigated PVL influences on CTh by including an appropriate control preterm cohort with no focal white matter abnormalities. Our PVL preterm sample showed thinner CTh in the temporal lobe, involving the medial and middle regions, when compared to full-term controls. This thinning is in keeping with a previous volumetric study using voxel-based morphometry [19], but some of the regions that showed volume decrements in that study, such as the postcentral, inferior parietal, middle frontal, and inferior temporal regions, did not show CTh reductions. Since gray matter volume depends on both CTh and surface area as explained above, and based on previous experimental observations [32], we speculate that PVL targeting of the neural precursor population at the periventricular germinal matrix may result in an undermining of the horizontal expansion of the developing cortex without an impairment of CTh. It has been proposed that volume is more closely related to surface area than cortical thickness [33]. On the other hand, thicker occipital lobe was seen bilaterally. This finding concurs with that of a study in adolescents, where subjects with and without PVL were analyzed together [20]. In fact, when we tried to isolate the effect of PVL by comparing preterms with and without PVL, we found that the presence of white matter lesions was associated with increased regional CTh in the pericalcarine and rostral middle frontal areas. Thicker cortices may reflect an atypical cerebral development with changes in the normal development curves. Thicker cortical regions in the same age range is a common finding in some neurodevelopmental disorders, such as autism [12], [13], and in absence epilepsy [34]. CTh is thought to be dependent on the processes of gray matter dendritic arborization and pruning [35] and the degree of myelinization at the gray/white matter interface [36]. Thus, disproportionate pruning, reduced neuronal/glial complement or immature development in specific cortical regions might be present in non-PVL preterm children, whereas an abnormal or delayed pruning program might occur in some PVL cortical areas. Further longitudinal studies with larger samples may help to clarify these CTh developmental trajectories in PVL preterm samples.

Intelligence scores have been correlated with gray matter volume in children born preterm without [15], [16] or with PVL [37]. In a recent study, a combination of total white matter volume together with the cross-sectional area of the corpus callosum explained most of the FIQ variance in preterm adolescents, irrespective of the extent of white matter injury [38]. In the present study, although the FIQ was significantly lower in the two samples of preterm children, we found a negative correlation only between FIQ and CTh in the full-term sample. Thus, higher FIQs were associated with cortical thinning. These findings are in line with previous reports that have also found this negative correlation in normal samples of children and adolescents [39], [40]. It has been suggested that cortical thinning reflects the process of synaptic pruning leading to improved efficiency of cerebral connectivity.

In addition to cognitive deficits, preterm samples also display behavioral problems compared to their term peers [41]. In our study, the main indexes for behavior did not differ between groups, as we and others have previously described [16], [42]. However, the attention subscale was found to be altered in PVL preterm children compared to full-term sample. Attention problems are a common finding in preterm children [2], [43]. Decrements in caudate nucleus volume [44], as well as in fractional anisotropy in the internal/external capsule and inferior/middle fascicles [45], have been related to attention deficits in preterm adolescents. Interestingly, we found that PVL preterm children showed the more pronounced thicker regions of the right hemisphere that are essential parts of the attentional network [46]. Indeed, frontal cortex and fronto-striatal structural and functional abnormalities are a common finding in children and adults with attention deficit hyperactivity disorder [47].

Regarding behavioral problems, we found that CTh in frontal areas correlated with internalizing and externalizing indexes. Internalizing problems imply higher scores in withdrawn, somatic complaints, and anxious/depressed scales, whereas externalizing problems reflect delinquent and aggressive behavior domains. We found that the higher the CTh values in the frontal areas, the more serious were the behavioral problems detected in childhood. In this respect, it is worth noting that the frontal cortical regions are involved in: (1) executive abilities (i.e. maintenance and redirection of attention, mental flexibility, shift in behavioral settings, etc.) dependent on the dorsolateral prefrontal circuit; (2) production of appropriate behaviors in response to social demands, which relate to the orbitofrontal circuit, and (3) generation of motivated behavior, which is controlled by the anterior cingulate gyrus [48]. In a recent study in healthy children, higher scores on the CBCL aggressive subscale were related to thinner anterior cingulate cortex [49]. However, our results showed a positive correlation between externalizing scores and CTh in cingulate cortex. As postulated for intelligence scores, the regional cortical thickness increments found in PVL preterm children, presumably due to defective synaptic pruning, may account for their worse scores in behavioral domains.

The main limitation of our study is its small sample size, which limits the statistical power. However, our study has some specific strengths. First, we selected appropriate control groups to analyze the independent impact of prematurity and PVL on CTh at school age. Second, we considered the characteristic trajectories of CTh regional growth curves occurring in the normal developing brain. Most brain regions attain the maximum peak of increment during childhood, followed by a decrement in adolescence and a subsequent stability in adulthood [11]. Consequently, all our analyses of global and regional CTh were controlled by age at examination. Finally, it is worth highlighting that CBCL is a parent-report form and might therefore be somewhat subjective.

This study provides evidence of the different patterns of CTh in childhood between a preterm cohort with no evidence of focal white matter abnormalities and a preterm cohort with brain injury such as PVL. In PVL preterm children, behavioral abnormalities are associated with thicker frontal areas, and attentional deficits are associated with increased CTh in several regions of the right hemisphere. Further research is needed to (1) determine developing CTh curves in PVL from the neonatal period onwards, (2) investigate whether the CTh differences observed in childhood persist into adolescence, and (3) establish whether they have a long-term impact on the cognitive and behavioral domains.
